# Diversity of Mycobiota in Spanish Grape Berries and Selection of *Hanseniaspora uvarum* U1 to Prevent Mycotoxin Contamination

**DOI:** 10.3390/toxins13090649

**Published:** 2021-09-13

**Authors:** Carolina Gómez-Albarrán, Clara Melguizo, Belén Patiño, Covadonga Vázquez, Jéssica Gil-Serna

**Affiliations:** Department of Genetics, Physiology and Microbiology, Faculty of Biology, University Complutense of Madrid, Jose Antonio Novais 12, 28040 Madrid, Spain; caroli13@ucm.es (C.G.-A.); claramel@ucm.es (C.M.); belenp@ucm.es (B.P.); covi@ucm.es (C.V.)

**Keywords:** metagenomics, biological control, *Hanseniaspora uvarum*, detoxification, aflatoxin B_1_, ochratoxin A

## Abstract

The occurrence of mycotoxins on grapes poses a high risk for food safety; thus, it is necessary to implement effective prevention methods. In this work, a metagenomic approach revealed the presence of important mycotoxigenic fungi in grape berries, including *Aspergillus flavus*, *Aspergillus niger* aggregate species, or *Aspergillus* section *Circumdati*. However, *A. carbonarius* was not detected in any sample. One of the samples was not contaminated by any mycotoxigenic species, and, therefore, it was selected for the isolation of potential biocontrol agents. In this context, *Hanseniaspora uvarum* U1 was selected for biocontrol in vitro assays. The results showed that this yeast is able to reduce the growth rate of the main ochratoxigenic and aflatoxigenic *Aspergillus* spp. occurring on grapes. Moreover, *H. uvarum* U1 seems to be an effective detoxifying agent for aflatoxin B_1_ and ochratoxin A, probably mediated by the mechanisms of adsorption to the cell wall and other active mechanisms. Therefore, *H. uvarum* U1 should be considered in an integrated approach to preventing AFB_1_ and OTA in grapes due to its potential as a biocontrol and detoxifying agent.

## 1. Introduction

Mycotoxins are secondary metabolites produced by a variety of filamentous fungi such as *Aspergillus* spp., *Penicillium* spp., *Alternaria* spp., and *Fusarium* spp. There are more than 500 types of mycotoxins, which are toxic to vertebrates and other animals after exposure [1]. Their ingestion may cause acute or chronic diseases depending on the dose and exposure time, and they have mutagenic, teratogenic, and/or carcinogenic properties [2]. Recent studies estimated that about 60–80% of global crops are contaminated by mycotoxins [3].

Spain is the third-largest wine producer worldwide and represents 14% of the global vineyard areas [4]. Several mycotoxins have been reported on grapes such as ochratoxin A (OTA), aflatoxin B_1_ (AFB_1_), patulin, fumonisin B_2_, alternariol, alternariol monomethyl ether, tenuazonic acid, and citrinin [5]. These mycotoxins are produced by different fungi reported on grapes such as *Aspergillus* spp., *Penicillium* spp., or *Alternaria* spp. [5]. Nevertheless, OTA remains to be the most commonly reported mycotoxin on grapes and their derivatives [6]. Due to its frequent occurrence and important toxic effects, OTA levels in grape products are strongly controlled by the current European legislation, which limits its concentration to 2 μg/kg in wine and grape juice [7]. Even though AFB_1_ on grapes is not as relevant as OTA and its presence on wine is not regulated yet, it cannot be ignored. Several authors have described its presence on grapes and wine from various regions, probably due to the wider distribution of its producing fungi due to climate change [8,9,10,11].

In this context, *Aspergillus* ochratoxigenic species are the predominant mycotoxin producers in grapes and their distribution is related to specific vineyard geographic locations and climatic conditions [5]. *Aspergillus carbonarius* and *Aspergillus niger* aggregate species are considered the most prevalent OTA producers in southern European regions and, specifically, in Spain [6,12,13]. Species belonging to the *Aspergillus* section *Circumdati* such as *Aspergillus westerdijkiae* or *Aspergillus steynii* have also been isolated from grapes, although their contribution to OTA contamination on grapes and derivatives is low [6]. The emerging occurrence of AFB_1_ is predominantly due to *Aspergillus parasiticus* and *Aspergillus flavus*, which are both occasionally isolated from grapes [11].

Control of mycotoxin contamination is focused on two strategies based on prevention of the development of mycotoxigenic fungi and/or detoxification [14]. The application of agricultural practices such as proper use of antifungals or controlling harvesting time is known to prevent the development of mycotoxigenic fungi in vineyards [5,15,16]. Currently, consumers demand harmless foodstuffs with an environmentally friendly production, which has led research to focus on the development of novel methods to prevent mycotoxin contamination [17]. Among these new approaches, biological control using antagonistic microorganisms such as yeasts or lactic acid bacteria is an emerging and very promising alternative [18,19].

Grape microbiota is extremely complex and includes a variety of bacteria, yeasts, and filamentous fungi. Among them, different mycotoxigenic species might occur but, in fact, these plant communities are also the source of most biocontrol agents described up to now [20]. Next-generation sequencing provides a useful tool to understand the complexity of grape microbiota and might be of interest in the search for new biological control agents (BCA) [20,21,22]. Yeasts represent the predominant group of microorganisms on the grape surface and are mainly represented by the yeast-like fungus *Aureobasidium pullulans*, oxidative or weakly fermentative ascomycetous (i.e., *Hanseniaspora* spp., *Candida* spp., *Metschnikowia* spp., or *Pichia* spp.), basidiomycetous populations (i.e., *Cryptococcus, Rhodotorula* spp., or *Sporobolomyces* spp.), and, in some cases, highly fermentative wine spoilage yeasts such a *Zygosaccharomyces* spp. or *Torulaspora* spp. [23].

Different biological methods have been proposed for controlling OTA in food products based on the application of yeasts, bacteria, or nontoxic fungi, which reduce fungal growth or decrease OTA levels [24]. In this context, epiphytic yeasts represent the most effective group of BCA due to their ability to colonize grapes and compete for space and nutrients with other microorganisms, such as mycotoxigenic fungi [15,25]. As mentioned before, epiphytic yeasts represent the largest community on grape berries and are highly adapted to this ecological niche; therefore, they might be considered effective biocontrol agents for their application in vineyards [22]. Accordingly, Bleve et al. [26] selected several yeasts that naturally occur on grapes (*Issatchenkia orientalis, Metschnikowia pulcherrima*, and *Candida incommunis*), which significantly controlled the fungal growth of ochratoxigenic isolates of *A. niger* and *A. carbonarius.* In addition, the ability of *A. pullulans* and *Lachacea thermotolerans* to reduce fungal growth and OTA biosynthesis by *Aspergillus* section *Nigri* species has also been reported [27,28].

Despite the application of control methods to avoid fungal growth, the presence of mycotoxins in foods cannot always be prevented. Moreover, the coexistence of several mycotoxins in the same product is a matter of concern because it is well-known that they have a cumulative and synergistic effect on toxicity and carcinogenicity, even at low levels of exposure [29,30]. Thus, it is necessary to develop new strategies of detoxification to eliminate mycotoxins from food.

Detoxification includes diverse methods (physical, chemical, or biological) that eliminate mycotoxins without generating toxic products or altering the nutritional value and organoleptic properties of foods [31,32]. In general, chemical detoxification is not allowed in the European Union due to biosafety concerns [14]. In the case of wine, OTA detoxification using physical adsorbents such as activated carbon during the fining process has been tested with positive results, although this treatment usually affects wine quality [33]. Considering the limitations of chemical and physical processes, biological detoxification is the most promising approach because it is safe from environmental and health perspectives, and it also maintains the organoleptic properties of foods [34]. These biological methods use microorganisms or their enzymes to degrade, transform, or adsorb mycotoxins and, therefore, avoid the toxic effects produced by the ingestion of contaminated food [35].

In this type of detoxification, it is indispensable that the microorganisms used as well as their degradation products are harmless to both humans and animals [35]. For this reason, current studies are focused on the selection of candidates for mycotoxin detoxification among probiotic microorganisms and other QPS (qualified presumption of safety) species [36]. Several microorganisms, including yeasts such as *Saccharomyces cerevisiae*, have shown mycotoxin detoxification properties in wines [19,37,38,39].

The aim of this work is to determine, through a metagenomic approach, the diversity of grape berry microbiota to study the occurrence of mycotoxigenic species and the presence of potential BCA candidates. The most relevant potential BCA, *Hanseniaspora uvarum* U1, was selected for subsequent studies to characterize its ability to control fungal growth of the main OTA- and AFB_1_-producing fungi found on grapes (*A. carbonarius*, *A. welwitschiae*, *A. steynii*, *A. westerdijkiae*, *A. flavus*, and *A. parasiticus*), as well as its detoxification potential relevant to these mycotoxins.

## 2. Results

### 2.1. Analysis of the Mycobiota of Grape Berries

A metagenomic approach was used to study the mycobiota present in grapes using the sequence of the ITS2 region of the rDNA. Rarefaction curves were constructed (Supplementary Figure S1) as a function of sampling depth. All curves reached the plateau and were close to saturation; therefore, the number of clones sequenced was sufficient to fully observe the richness of the samples. The complete results of relative abundance at genus level are included in Figure S2.

Alpha-diversity was studied using Shannon, Chao1, and inverse Simpson indexes (Table 1). No statistically significant differences were found in any diversity index when the effect of the location or the vineyard management was evaluated; all samples analyzed showed similar richness and evenness. The high levels of Chao1 and Shannon indexes are related to rich and uniform fungal communities, and inverse Simpson values indicate no clear dominance of one or more species. Although no differences were reported, M2 showed lower values of the three indexes evaluated, possibly due to the high predominance of *Penicillium* spp., representing 98% of the total diversity.

Different mycotoxigenic species were detected in grape berries and their distribution widely varies among samples. *Aspergillus* section *Nigri* species were frequently found but represented very different percentages of the total fungal diversity, and *A. carbonarius* was not detected in any sample. It is important to highlight the presence of *A. flavus* in all but one grape sample, indicating a potential risk of aflatoxin contamination. Moreover, potential ochratoxigenic *Aspergillus* section *Circumdati* species (*A. westerdijkiae* and *A. steynii*) were also detected in two of the eight samples. Sample M2 was the only one not contaminated by any potential toxigenic species.

The presence of species that have been reported as potential biocontrol agents against mycotoxigenic fungi was also evaluated. Most of the diversity in all samples was represented by the yeast-like fungus *Aureobasidium pullulans*, with up to 54% of the total diversity. The yeast *Hanseniaspora uvarum* was also detected in 75% of the grape samples and, in some cases, represented high percentages of the diversity of the total mycobiota. Other significant yeasts detected that had been described previously as potential biocontrol agents were *Debaryomyces hansenii*, *Lanchacea* sp., and *Candida* sp.

Considering that sample M2 was not contaminated by any mycotoxigenic species, it was selected for the isolation of potential biocontrol agents since the yeasts that occur there might influence the occurrence of these fungi. The most frequently isolated yeast from rose bengal chloramphenicol agar plates was *Hanseniaspora uvarum* and the isolate U1 was selected for subsequent studies. The identification was confirmed using sequencing of the D1–D2 region. The sequence was deposited in the NCBI database (accession number OK039223).

### 2.2. Biocontrol

**Figure 1:**
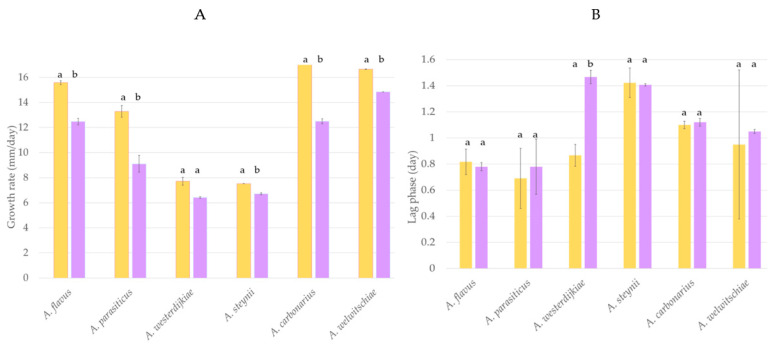
Growth rate (**A**) and lag phase (**B**) of *A. flavus*, *A. parasiticus*, *A. westerdijkiae*, *A. steynii*, *A. carbonarius*, and *A. welwitschiae* cultured in control CYA plates (yellow) and supplemented with *H. uvarum* U1 (purple). Each value is the mean of two replicates. Thin vertical bars represent the standard error of the corresponding data. Groups with the same letter are not significantly different (*p* > 0.05).

The potential of *H. uvarum* U1 to prevent fungal growth was evaluated using *A. carbonarius*, *A. welwitschiae*, *A. westerdijkiae*, *A. steynii*, *A. flavus*, and *A. parasiticus*. Results regarding the influence of *H. uvarum* U1 on the lag phase and growth rate of each fungus are shown in Figure 1. The growth rate of most fungi tested was significantly reduced when *H. uvarum* U1 was added into the CYA plates. The reduction percentages were 34, 31, and 19% in the case of *A. carbonarius*, *A. parasiticus*, and *A. flavus*, respectively, whereas the growth rates of *A. steynii* and *A. welwitschiae* were reduced by 11%. The growth rate of *A. westerdijkiae* was not affected by the presence of *H. uvarum* U1, but its lag phase was extended by 75%, suggesting that the potential BCA can control fungal growth in the first steps of the cycle (Figure 1 and Figure S3).

### 2.3. Detoxification Ability of Aflatoxin B_1_ and Ochratoxin A by Hanseniaspora uvarum U1

First, the effect of mycotoxins on yeast development and viability was evaluated. For this purpose, the growth of *H. uvarum* U1 was measured at a different pH (3.0, 5.5, and 7.0) at 630 nm in the presence and absence of the analyzed concentration of aflatoxin B_1_ (AFB_1_) and ochratoxin A (OTA). In addition, the effect of the mycotoxin solvent (methanol) on *H. uvarum* U1 growth was evaluated. Table 2 shows viable cell (VC) absorbance values, which correspond to the growth of *H. uvarum* U1. Yeast growth at pH 3.0, 5.5, and 7.0 was not affected by the amount of AFB_1_, OTA, or methanol used (*p*-value for pH 3.0 = 0.377; *p*-value for pH 5.5 = 0.539; *p*-value for pH 7 = 0.967). Therefore, neither mycotoxins nor methanol reduced the growth of *H. uvarum* U1.

The ability of viable cells (VC) and heat-inactivated cells (HIC) of H. uvarum U1 to remove AFB1 and OTA at different pH (3.0, 5.5, and 7.0) was evaluated by ELISA. As shown in Table 3, H. uvarum U1 cells were able to significantly reduce AFB1 and OTA at all pH and treatments tested. In general, the highest reduction values were found for AFB1. The greatest AFB1 removal was observed at pH 5.5 (VC = 99.08%; HIC = 97.68%). There were significant differences in the removal percentage of AFB1 between VC and HIC H. uvarum U1 when incubated at pH 3 (VC = 93.68%; HIC = 83.84%) and pH 7.0 (VC = 98.29%; HIC = 95.78%), and VC was more efficient at detoxifying AFB1. These differences between VC and HIC were not observed at pH 5.5.

In the case of OTA, the maximum removal ability of VC was observed at pH 7.0 (82.96%), whereas for HIC, the highest percentage of reduction occurred at pH 5.5 (79.87%). Significant differences were found at pH 3.0, and the highest reduction percentage was found in the case of VC.

In general, the percentage reduction of AFB1 and OTA was higher in viable cells than in HIC. The lowest percentages of AFB1 and OTA detoxification occurred at pH 3.0 and with inactivated cells.

## 3. Discussion

Ochratoxin A (OTA) is considered the most important mycotoxin on grapes and wines [6]. However, new weather conditions as a result of climate change favor the growth of aflatoxin B_1_-producing fungi and, therefore, AFB_1_ is an emerging mycotoxin in grapes and grape products [11]. The impact of these mycotoxins on health and the economy has led to an increase in the search for effective measures to reduce mycotoxigenic fungi. Although the effectiveness of chemical methods to control mycotoxigenic fungi has been established, there is a growing public demand and deep concern within the European Union to find safer and more environmentally friendly alternatives to avoid mycotoxins in foodstuffs [40]. Biological control is now considered a suitable approach to prevent fungal pathogens on grapes in both preharvest and postharvest [41,42] and has even been proposed as an effective measure to reduce the development of mycotoxigenic fungi [16,24]. Among potential biological control agents (BCA), yeasts present particular characteristics that make them considered the most adaptable organisms for use in grapes [22].

The metagenomic approach performed in this work was useful for studying the fungal mycobiota on grape berries and, specifically, the occurrence of potential mycotoxigenic fungi. Although the presence of aflatoxigenic species has not been studied systematically, there are several reports available that describe the presence of *A. flavus* and *A. parasiticus* on grapes [11]. In this work, *A. flavus* was consistently detected in almost all samples analyzed, which might be related to an important risk of contamination by aflatoxins. Several articles have pointed out that *A. flavus* is an emerging problem mainly in temperate, southern regions of Europe, since it is able to grow and produce aflatoxins under these conditions [43]. Therefore, this work might be a reflection of the expansion of *A. flavus* distribution across Europe and its increased occurrence in unconventional matrices.

Surprisingly, *A. carbonarius* was not detected in any of the samples despite being reported for a long time as the predominant ochratoxigenic fungus in grapes, mainly in Mediterranean vineyards [5,6,44]. The findings of this work might agree with those reported by Cervini et al. [45], who demonstrated that the climate change scenario predicted for this region might affect *A. carbonarius* development and its ability to produce OTA in grapes. However, the risk posed by OTA contamination should not be disregarded since other important producers included in the section *Circumdati* such as *A. steynii* and *A. westerdijkiae* have been detected in several samples. *Aspergillus niger* aggregate species were also present in some grape samples and, although their importance as OTA producers is discussed, their presence might lead to FB_2_ contamination promoted by the new forecast conditions of the climate change scenario [10].

The metagenomic analysis of fungal microbiota also allowed the identification of potential biocontrol agents among the epiphytic yeasts. The results regarding yeast occurrence on grape berries are similar to those previously reported by other authors [23]. *Aureobasidium pullulans* yeast-like fungus was by far the most representative group of fungal microbiota. This is an indicator of its ability to establish and dominate in this ecosystem, which is one of the required characteristics of a potential biocontrol agent. However, *A. pullulans* has been related to human infections in immunocompromised patients and it is not included in the GRAS and QPS lists. Therefore, its potential use in the environment is not advisable without performing prior in-depth studies [46,47]. Moreover, this yeast-like fungus is not included in the GRAS and QPS lists, and its inclusion is unlikely due to its possibility of being a human pathogen. The second most frequent yeast in grape berries was *Hanseniaspora uvarum*. Several works have reported that this yeast can occupy 50% of the total population on grapes [48,49]. Non-*Saccharomyces* yeasts such as *H. uvarum* play an important role in the early stages of fermentation as they can release hydrolytic enzymes that improve the aromatic and sensory properties of wines [49,50]. Moreover, *H. uvarum* has been previously proposed as an effective BCA against different fungal pathogens in grapes included in the genera *Aspergillus*, *Penicillium*, *Alternaria*, and *Botrytis* [51,52,53,54,55]. Therefore, this species was considered for isolation from grape samples since it could be a suitable candidate as a biocontrol agent against mycotoxigenic fungi on grapes.

*Hanseniaspora uvarum* U1 was isolated from grapes sampled in a conventionally managed vineyard from Spain. Although *H. uvarum* was detected in all samples, the grapes sampled in this vineyard (M2) were not contaminated by any mycotoxigenic species and, therefore, it could be possible that some effective BCA was present and controlling fungal development. The presence of *H. uvarum* U1 was demonstrated to affect the fungal growth of important species frequently occurring in grapes that are able to produce both aflatoxins and OTA. *Aspergillus westerdijkiae* was the only fungi not significantly affected by the presence of *H. uvarum*. However, a potential BCA for controlling mycotoxins in foodstuffs might not only affect fungal growth and its ability to colonize the product but also inhibit or reduce mycotoxin biosynthesis [56]. Prendes et al. [55] recently reported that though *H. uvarum* can stimulate *Alternaria alternata* growth, it also produces high inhibition rates of mycotoxin production. Therefore, further studies are necessary to unravel the effect of *H. uvarum* U1 on the ability to produce mycotoxins by these *Aspergillus* species.

Although controlling fungal growth is considered the most effective method for avoiding mycotoxins, in some cases and conditions, it cannot be implemented. In this context, some yeasts present desirable characteristics for application as biological detoxifying agents by adsorption or degradation of mycotoxins [19]. Therefore, in this work, the potential of *H. uvarum* U1 to detoxify AFB_1_ and OTA was also tested. Some mycotoxins present cytotoxic effects, and some yeasts can be highly sensitive; therefore, yeast growth and survival should be tested before the optimization of a detoxifying agent [19]. At the doses tested, neither AFB_1_ nor OTA affected *H. uvarum* development, which is an advantage for the future application of viable cells for mycotoxin decontamination.

To our knowledge, there is only one previous report that describes the ability of this yeast to detoxify mycotoxins [57]. In a preliminary study, *H. uvarum* U1 showed a high mycotoxin detoxification ability since it was able to remove 99% and 83% of AFB_1_ and OTA, respectively.

Mycotoxin removal by microorganisms can occur by biodegradation and/or by adsorption to the cell walls [19,58,59]. To determine its application in food products, it is essential to evaluate whether one or both mechanisms are involved in the elimination of AFB_1_ and OTA by *H. uvarum* U1; therefore, experiments were performed using viable and heat-inactivated cells. AFB_1_ and OTA were efficiently removed by both cells, which indicates that adsorption may be one of the mechanisms used by *H. uvarum* U1 to detoxify these mycotoxins. Adsorption is a physical process that does not depend on the viability or physiological state of the cell and can also occur in inactivated cells. However, it has been claimed that heat treatment might result in an increased exposure of binding sites on nonviable cells [60], although this effect has not been observed in the case of *H. uvarum* U1. In general, viable cells were more efficient in mycotoxin removal than heat-inactivated yeasts and, therefore, other mechanisms might be involved in *H. uvarum* U1′s detoxification capacity.

Subsequently, in this work, the ability of *H. uvarum* U1 to detoxify AFB_1_ and OTA at different pH was analyzed. Several authors described pH as an important factor involved in mycotoxin adsorption, especially when electrostatic interactions are involved [61]. Under these circumstances, pH can affect the degree of ionization of mycotoxins and the charge distribution of microorganism wall components. AFB_1_ is a nonionizable molecule and its adsorption should not be drastically affected by pH [61]. The results of this work indicate a lower reduction of AFB_1_ at pH 3.0, although the differences in adsorption with respect to other pH are not as pronounced as those observed in OTA. In the case of OTA adsorption by yeasts, several studies reported that acidic conditions increase detoxification values when compared to alkaline pH [62,63]. The acidic character of OTA (pKa 4.4) causes the protonated and uncharged forms to be present in acidic solutions (pH < 4.0), which is supposed to enable its binding to yeast wall components such as glucans [64]. These components provide numerous binding sites for mycotoxin adhesion. Glucans represent up to 60% of the cell wall of *Saccharomyces cerevisiae*, and, therefore, most research has been focused on them [60,65,66]. However, the ability of *H. uvarum* U1 to detoxify OTA was strongly reduced in acidic conditions, which might be due to its unusual wall composition, being mostly enriched with proteins [67]. Moreover interactions involving basic amino acids are highly pH-dependent, which may affect in some way the OTA binding to the cell wall of *H. uvarum* when subjected to such an acidic pH

The adsorption process also depends on the three-dimensional conformation of the cell wall components. Yiannikouris et al. [68] described a higher affinity of AFB_1_ for the conformation of binding sites offered by β-d-glucans compared to OTA. Although the percentage of glucans in *H. uvarum* cell wall is low, this fact could explain why, under the same pH conditions, elimination is higher in the case of AFB_1_.

In this study, viable *H. uvarum* U1 cells showed a greater ability to reduce AFB_1_ and OTA than heat-inactivated cells. These results indicate that the detoxification process also involved an active mechanism, such as degradation. Angioni et al. [57] described the ability of *H. uvarum* to degrade OTA but did not unravel the pathway involved. Other articles reported that *Trichosporon*, *Rhodotorula*, *Cryptococcus*, and *Aureobasidium pullulans* are able to degrade OTA by cleavage of the amide bond [69,70]. Hydrolytic enzymes such as carboxypeptidases, ochratoxinases, proteases, and lipases have been identified in most of the described microorganisms as agents capable of degrading OTA into less-toxic by-products [35,71]. The highest OTA removal by *H. uvarum* U1 was found at conditions in which these enzymes have maximum hydrolytic activity (pH between 6.0 and 7.5) [71,72] and, therefore, a degradation mediated by these hydrolytic enzymes might be involved in the detoxification ability of this yeast. Recent work reported that strains of *Pichia occidentalis*, *Candida sorboxylosa*, and *Hanseniaspora opuntiae* isolated from kombucha are able to degrade AFB_1_ into less-toxic products [73]. Furthermore, Li et al. [74] postulated the mechanism by which *Candida versatilis* CGMCC 3790 degrades AFB_1_ and found that the optimal degradation condition was at pH 5.0. These results agree with those observed in *H. uvarum* U1, although more research is needed to characterize the degradation process.

Vanhoutte et al. [75] established the ideal features of a detoxifying agent that include the following: (I) the process should be fast and efficient, (II) the process should occur on more than one mycotoxin, (III) the agent must not be pathogenic, (IV) the resulting degradation products must not be toxic, and (V) the degradation must occur under conditions that are relevant to the contaminated matrix. *Hanseniaspora uvarum* U1 accomplishes most of these tasks, since it is able to remove both AFB_1_ and OTA very effectively in a short time and, additionally, it is qualified as a QPS agent [76].

## 4. Conclusions

This work presents an integrated approach to prevent AFB_1_ and OTA in grapes. The study of the mycobiota by a metagenomic approach allowed the characterization of both mycotoxigenic risk and potential biocontrol agents. Moreover, *Hanseniaspora uvarum* U1 was demonstrated to be a promising biocontrol agent since it is capable of controlling fungal growth in different aflatoxigenic and ochratoxigenic species. Moreover, this yeast is able to detoxify AFB_1_ and OTA extracts in a wide range of conditions, and the most probable detoxification pathway includes both adsorption to the cell wall and an active mechanism.

## 5. Materials and Methods

### 5.1. Sample Collection

Samples were collected in four locations in Spain (Toledo, M1 and M2; Valencia, M3 and M4; Burgos, M5 and M6; Madrid, M7 and M8), and two vineyards were selected from each site to include both ecological and conventional practices (even and odd sample number, respectively). For each vineyard, the samples were collected at random, taking one grape cluster every 5 m along the plot, reaching a total of approximately 3 kg of grapes. At the laboratory, all samples were manually destemmed, mixed, and divided into lots of 100 g. Subsequently, the subsamples were processed using a lyophilizer (Cryodos, Telstar, Madrid, Spain) and stored at 4 °C until analysis.

### 5.2. DNA Extraction and Metagenomic Analysis

DNA isolation from lyophilized grapes was performed using the DNeasy Plant Mini Kit (QIAgen, Hilden, Germany) following the manufacturer’s instructions. Three DNA extractions were carried out for each sample and were subsequently mixed and concentrated using a vacuum concentrator (Concentrator plus, Eppendorf, Hamburg, Germany).

For metagenomic analysis of fungal microbiota, the ITS2 region of ribosomal DNA was selected and amplified using ITS3 and ITS4 universal primers [77]. Amplicon libraries were prepared using Herculase II Fusion DNA Polymerase Nextera XT Index Kit V2 (Illumina, Foster City, USA) and sequenced on a MiSeq Illumina Platform in Macrogen facilities (Macrogen Inc., Seoul, Korea).

Data processing and analysis were performed using different bioinformatic software, which processed and analyzed the obtained sequence data. Preliminary processing of the data was performed using FLASH 1.2.11 [78] to merge paired-end reads. Subsequently, CD-HIT-OTU 4.5.5 [79] was used to filter and trim raw sequences, which were then clustered into operational taxonomical units (OTU). Taxonomy assignments were performed using the UNITE database [80]. Further analyses were carried out using QIIME v. 1.8 [81], and diversity indexes such as alpha diversity, Shannon, and Chao1 were estimated.

### 5.3. Isolation and Identification of Hanseniaspora uvarum Isolated from Grapes

The source of yeast isolation was sample M2 from Toledo taken in a conventionally managed vineyard. This sample was selected due to the absence of mycotoxigenic fungi, which might be related to the presence of established microorganisms that can effectively compete in this substrate. For this purpose, surface-disinfected grapes were cultured on rose bengal chloramphenicol agar plates (Pronadisa, Madrid, Spain) for 48 h at 28 °C to recover the maximum number of yeast species present in the grapes. Isolated colonies were then selected and reisolated on potato dextrose agar plates (PDA, Pronadisa, Madrid, Spain). All yeasts were stored in 15% glycerol (Fisher Chemical, Loughborough, UK) at −80 °C until required.

Before DNA extraction, the isolated yeasts were cultured in 20 mL of potato dextrose broth (PDB) (Pronadisa, Madrid, Spain) at 28 ± 1 °C in an orbital shaker SK-0330-PRO (140 rpm) (Labolan, Esparza, Spain) in darkness. Subsequently, 1 mL of the culture was centrifuged, the supernatant removed, and DNA extraction was performed. Genomic DNA extraction from yeast isolates was carried out using a protocol described elsewhere [34].

The identification was performed by sequencing the variable D1–D2 domain of the 26S subunit region using the protocols described by Kurtzman and Robnett [82]. PCR assays were performed in an Eppendorf Mastercycler Gradient (Eppendorf, Hamburg, Germany). All amplification reactions were carried out in a final volume of 25 μL, containing 100 ng of sample DNA, 1 μL of each primer (20 μM) (Metabion, Planneg, Germany), and 12.5 μL NZYTaq II 2× Green Master Mix (NZYTech, Lisbon, Portugal). PCR-amplified products were visualized in a 1.5% agarose gel electrophoresis (Pronadisa, Madrid, Spain) using 1× TAE buffer (Tris-acetate 40 mM and EDTA 1.0 mM) and 3 μL of Green Safe Premium (1 μg/mL) (NZYTech, Lisbon, Portugal). The NZYDNA Ladder V (NZYTech, Lisbon, Portugal) was used as a molecular size marker. The electrophoresis was performed at 80 V for 25 min and then visualized under UV light (ETX-20-M, Vilber Lourmat, France).

The amplification products were purified with a NZYGelpure Kit (NZYTech, Lisbon, Portugal). Sequencing was carried out on an ABI PRISM 3730XL DNA sequencer (Applied Biosystems, Foster City, USA) according to the manufacturer’s instructions in Macrogen facilities (Madrid, Spain). All amplification products were sequenced in both directions.

The sequences were assembled using the UGENE v33.0 package. Sequences were compared with those deposited on the NCBI nucleotide database using Blast to extend their identification to species level.

### 5.4. Biological Control Using Hanseniaspora uvarum U1

Aflatoxin-producing strains of *A. flavus* (A23) and *A. parasiticus* (A19) as well as OTA-producing strains of *A. carbonarius* (350), *A. westerdijkiae* (AODP 16-1), *A. welwitschiae* (N5-M16), and *A. steynii* (3.53) were selected to test the potential of *H. uvarum* U1 to reduce fungal growth in vitro. Fungal strains were cultured on potato dextrose agar (PDA) and incubated at 28 °C for five days. Subsequently, spore suspensions were prepared in sterile saline solution (9 g/L sodium chloride), and the concentration was determined using a Thoma counting chamber (Marienfeld, Lauda-Königshofen, Germany) and adjusted to a final concentration of 10^6^ spores/mL. *Hanseniaspora uvarum* was cultured in PDA plates for 48 h at 28 °C and a cell suspension was prepared following the same method used for spore suspensions.

The effect of the presence of yeast on fungal growth was evaluated in CYA medium (45.5 g/L of modified Czapek–Dox agar (Pronadisa, Madrid, Spain) and 5 g/L of yeast extract (Pronadisa, Madrid, Spain)). *Hanseniaspora uvarum* U1 was added to the medium to obtain a final concentration of 10^3^ cells/mL. The same amount of saline solution was included in control plates instead of *H. uvarum* U1. Both plates were inoculated with 2 µL (4 mm diameter) of each fungal spore suspension in the center of the plate. Fungal colony diameter was measured daily in two directions until the surface of the control plates was fully colonized, which took 5 days for *A. carbonarius*; 6 days for *A. flavus*, *A. parasiticus,* and *A. welwitschiae*; and 9 days for *A. westerdijkiae* and *A. steynii*. Growth parameters were calculated from a linear model by plotting diameter (mm) against time (day). The lag phase (days prior to mycelial growth) and growth rate (mm/day) were calculated both for control plates and *H. uvarum*-supplemented ones.

### 5.5. Study of the Detoxification Capacity of Ochratoxin A and Aflatoxin B1 by Hanseniaspora uvarum U1

#### 5.5.1. Preparation of Yeast and Toxin Extracts

Stock solutions of OTA and AFB_1_ (Sigma-Aldrich, Darmstadt, Germany) were prepared in 5 mL of methanol, to a final concentration of 200,000 μg/L. These initial stock solutions were diluted up to 0.5 μg/L and 1 μg/L, respectively. Concentrations were standardized to add 2.5 μL of AFB_1_ and OTA to each well.

*Hanseniaspora uvarum* U1 suspensions were prepared as mentioned above to a final concentration of 1.5 × 10^8^ CFU/mL. These resulting suspensions constituted the viable cells (VC).

#### 5.5.2. Detoxification Assays

The analysis of the detoxification capacity of *H. uvarum* U1 for AFB_1_ and OTA was performed in 96-well polystyrene microplates (Corning^TM^, USA). The viable cells experiment included the following conditions: (I) A growth control—25 μL of the *H. uvarum* suspension; 200 μL of potato dextrose broth (PDB) (Pronadisa, Madrid, Spain) at pH 3.0, 5.5, or 7.0, and 25 μL of 0.9% *w*/*v* NaCl solution to complete the volume. (II) A test sample containing 25 μL of the yeast suspension; 200 μL of PDB at pH 3.0, 5.5, or 7.0; 2.5 μL of AFB_1_ or OTA; and 22.5 μL of saline solution. (III) Methanol control for toxicity, which included the same components as (i), replacing the 2.5 μL of mycotoxin with methanol. (IV) Mycotoxin control with 200 μL of PDB medium at pH 3.0, 5.5, or 7.0; 2.5 μL of AFB_1_ or OTA; and 47.5 μL of saline solution. All samples and controls described before were analyzed by duplicate. The microplates were incubated for 48 h at 30 °C without shaking and yeast growth was then evaluated by measuring the turbidity in each well at 630 nm with a plate reader (Dutscher, Bernolsheim, France). Subsequently, samples and controls containing mycotoxins were filtered using 0.22 μm filters (Fisherbrand, Thermo Fisher Scientific, Madrid, Spain), and the filtered products were stored in 1.5 mL Eppendorf tubes at -20 °C until mycotoxin quantification by ELISA.

A similar approach was performed to evaluate the ability of heat-inactivated cells (HIC) of *H. uvarum* U1 to detoxify OTA and AFB_1_. As the first step, 25 μL of the *H. uvarum* U1 suspension was incubated in multi-well plates with 200 μL of PDB and 25 μL of saline solution for 48 h at 30 °C. In this way, there was the same number of cells in both experiments. After incubation, mixtures were inactivated by heat treatment at 121 °C for 20 min in an autoclave and, subsequently, 2.5 μL of AFB_1_ and OTA extracts were added to each one. These samples were incubated for 30 min at room temperature and then filtered. The filtered products were finally stored at −20 °C until quantification by ELISA.

#### 5.5.3. Quantification of Mycotoxins by ELISA

Quantification of AFB_1_ and OTA was performed using the RIDASCREEN^®^ Aflatoxin B1 30/15 Art. No. R1211 and RIDASCREEN^®^ Ochratoxin A 30/15 Art. No. R1311 (R-Biopharm, Darmstadt, Germany), respectively, following the manufacturer’s instructions. These kits are based on a competitive ELISA assay, in which the absorbance measured is inversely proportional to the mycotoxin concentration of the sample. The resulting color reaction was measured at 450 nm with a plate reader (Dutscher, Bernolsheim, France).

A six-point standard curve was prepared using samples with different concentrations of AFB_1_ (0; 1; 5; 10; 20; 50 μg/L) (%(B/B0) = −35,291 log(concentration) + 76,952; R^2^ = 0.9884) and OTA (0; 0.05; 0.1; 0.3; 0.9; 1.800 μg/L) (%(B/B0) = −50,442 log(concentration) + 167.98; R^2^ = 0.9607) included in each RIDASCREEN^®^ ELISA kit. First, the percentage of absorbance (% (B/B0)) was calculated by the following formula:

% (B/B0) = (Standard absorbance or sample (B)/Absorbance of blank (B0)) × 100

The absorbance data obtained in the detoxification test were interpolated from the line to obtain the concentration value of AFB_1_ and OTA present in the samples after incubation. The standard line was made using Microsoft Excel^®^ software (Microsoft Corporation, Washington, USA).

### 5.6. Statistical Analysis

Statistical analyses were performed with StatGraphics Centurion XVII V.17.2.04 software (Statpoint Technologies Inc., Warrenton, VA, USA). Normality and homoscedasticity of the data were tested by the Shapiro–Wilk and Bartlett tests. In the case of diversity indexes, percentage of mycotoxin reduction in detoxification assays, and growth absorbance values, an analysis of variance (ANOVA) was performed followed by Fisher’s LSD post hoc test for checking differences among group means. For lag phase and growth rate in biological control experiments, a statistical analysis was performed independently for each mycotoxigenic strain using Student’s t-test. In all cases, the significance level was set at *p* < 0.05.

## Figures and Tables

**Table 1:** Metagenomic analysis of the mycobiota in grape berries. The percentage of abundance related to the total mycobiota of toxigenic fungi and yeasts that could be used as potential biological control agents are shown. Diversity indexes for each sample are also included.

	M1	M2	M3	M4	M5	M6	M7	M8
**Diversity Indexes**								
Chao1	53	15	63	86	37	56	50	60
Shannon	2.519	0.908	2.671	1.954	2.016	2.244	1.797	2.462
Inverse Simpson	0.760	0.380	0.766	0.525	0.747	0.656	0.574	0.689
**OTU Number**								
Genera	34	10	45	45	23	37	33	33
**Yeast (Total)**	53.39	1.36	45.89	31.51	28.17	57.66	51.15	33.99
*Aureobasidium pullulans*	37.36	1.32	44.75	23.55	27.89	54.33	48.48	33.37
*Hanseniaspora uvarum*	15.55	0.04	0.11	1.98	0.003	-	-	0.01
*Lanchacea* spp.	0.31	-	-	1.07	-	-	-	-
*Debaryomyces hansenii*	-	-	-	0.07	-	0.03	-	-
*Candida* spp.	-	-	-	-	-	0.02	0.16	-
**Potential Toxigenic Fungi (Total)**	30.44	-	0.01	0.99	0.013	0.18	0.36	3.11
Uniseriate black *Aspergillus*	28.51	-	-	-	0.01	-	-	0.09
*Aspergillus niger* aggregate	1.91	-	-	-	-	0.01	-	2.48
*Aspergillus* section *Circumdati*	-	-	-	0.07	-	-	-	0.13
*Aspergillus flavus* related	0.02	-	0.01	0.92	0.003	0.17	0.36	0.41

**Table 2:** Absorbance values at 630 nm after culturing *Hanseniaspora uvarum* U1 on PDB at different pH (3.0, 5.5, and 7.0) and treatments (methanol, AFB_1_, OTA) after 48 h at 30 °C. Results are mean values ± standard deviations of two samples. For each pH, comparisons were performed between different treatments. There were no significant differences between control and treatments (methanol and mycotoxins) in any case (*p* < 0.05).

Treatment	pH 3	pH 5.5	pH 7
Control	1.285 ± 0.007	1.380 ± 0.012	1.310 ± 0.043
Methanol	1.316 ± 0.017	1.356 ± 0.025	1.310 ± 0.002
AFB_1_	1.298 ± 0.015	1.351 ± 0.011	1.310 ± 0.013
OTA	1.272 ± 0.010	1.380 ± 0.009	1.288 ± 0.011

**Table 3:** Percentages of mycotoxin reduction by viable cells (VC) and heat-inactivated cells (HIC) of *H. uvarum* U1 obtained at different pH (3.0, 5.5, and 7.0) after 48 h of incubation at 30 °C and 30 min. Results are mean values ± standard deviations of two samples. Comparisons were performed for each mycotoxin independently. Different letters (a–c) in the same column (pH) indicate a significant effect among control, VC, and HIC. Different letters (y–z) in the same row (treatment) indicate a significant effect of pH (*p* < 0.05).

	Mycotoxin Reduction (%)					
Treatment	AFB_1_			OTA		
	3.0	5.5	7.0	3.0	5.5	7.0
Control	0 ± 0 a	0 ± 0 a	0 ± 0 a	0 ± 0 a	0 ± 0 a	0 ± 0 a
VC *H. uvarum* U1	93.68 ± 1.22 by	99.08 ± 0.10 bz	98.29 ± 0.63 bz	46.27 ± 0.01 by	81.09 ± 0.05 bz	82.96 ± 1.14 bz
HIC *H. uvarum* U1	83.84 ± 1.99 cy	97.68 ± 1.10 bz	95.78 ± 0.50 cz	24.23 ± 6.15 cy	79.87 ± 0.36 bz	78.64 ± 0.69 bz

## Data Availability

Raw data are available upon request, please contact the corresponding author.

## Supplementary Materials

The following are available online at https://www.mdpi.com/article/10.3390/toxins13090649/s1, Figure S1, rarefaction curves. Figure S2, relative abundance of the main fungal genera in grape samples, based on the metagenomic analysis of ITS2 sequences. Figure S3, colony growth of *A. parasiticus* and *A. carbonarius* on control plates and when incubated in the presence of *H. uvarum* U1.
