# Supplementary material for: Diversity of Mycobiota in Spanish Grape Berries and Selection of Hanseniaspora uvarum U1 to Prevent Mycotoxin Contamination
Source: Toxins (Basel). 2021 Sep 13;13(9):649. doi: 10.3390/toxins13090649 (PMC8473298; doi:10.3390/toxins13090649)
Supplement: Supplementary file 1 [file toxins-13-00649-s001.zip › toxins-1316971 supplementary.pdf]

toxins

Supplementary Materials: Diversity of Mycobiota in Spanish Grape Berries and Selection of *Hanseniaspora uvarum* U1 to Prevent Mycotoxin Contamination

Carolina Gómez-Albarrán, Clara Melguizo, Belén Patiño, Covadonga Vázquez and Jéssica Gil-Serna

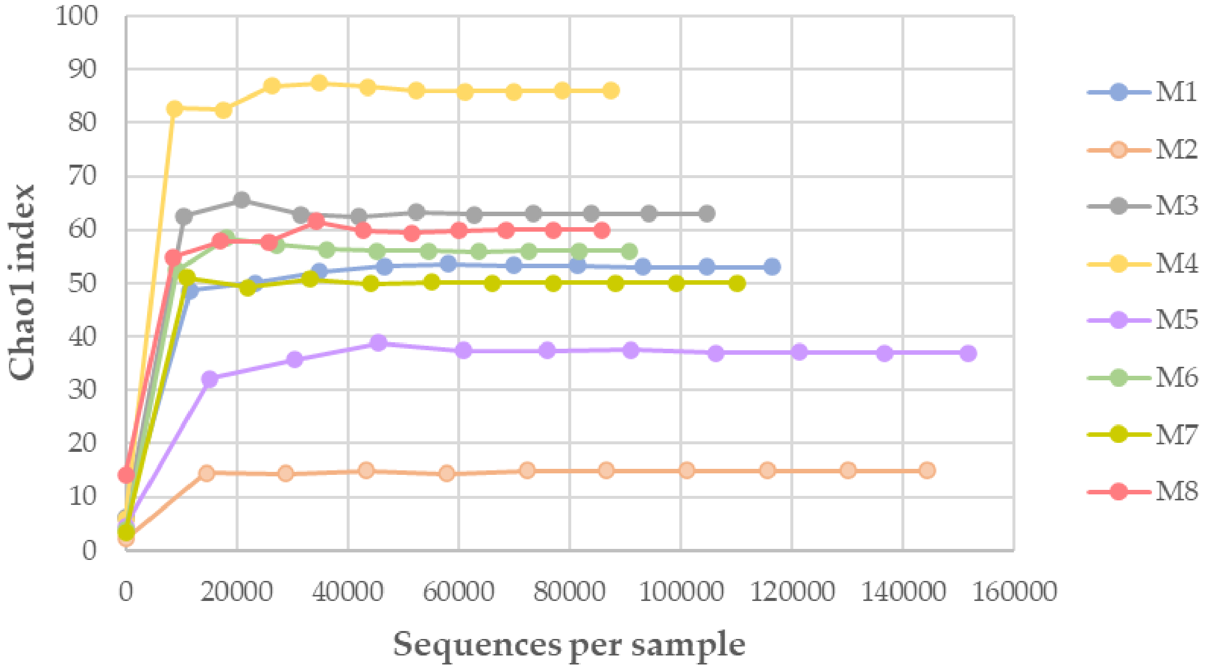

Figure S1. Rarefaction curves. Chao1 index is plotted against the sequences obtained on each sample.

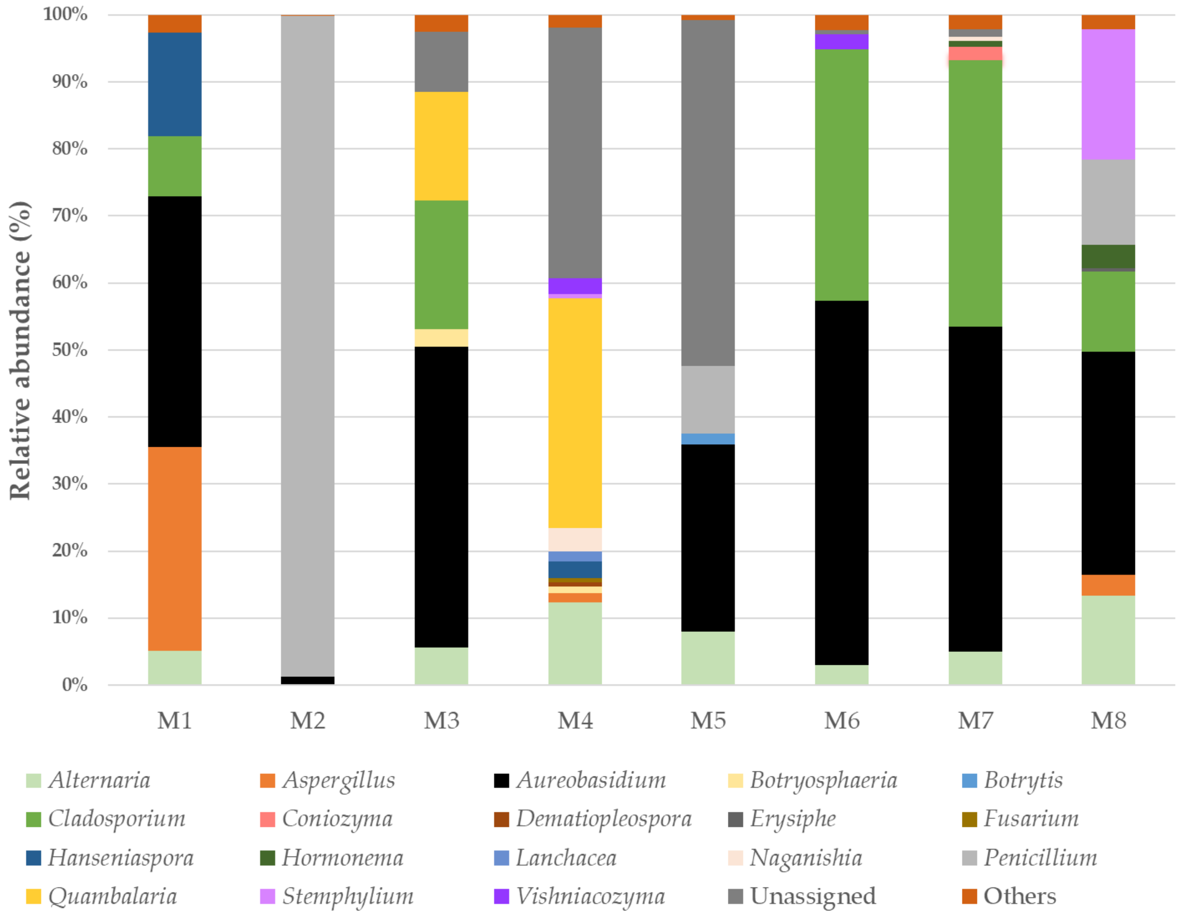

Figure S2. Relative abundance of the main fungal genera in grape samples, based on the metagenomic analysis of ITS2 sequences. Only the taxa with a taxonomic attribution at the level of genus which appear at least with abundance higher than 0.5% are included. The rest genera are grouped as "Others".

Control + *H. uvarum* U1

A. parasiticus

A. carbonarius

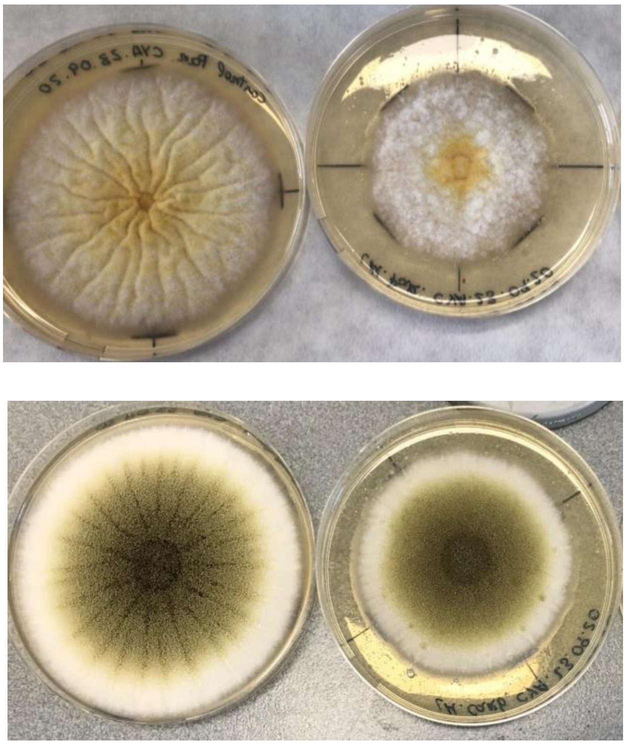

Figure S3. Colony growth of *A. parasiticus* (above) and *A. carbonarius* (below) on control plates and when incubated in the presence of *H. uvarum* U1.
